# Long non-coding RNA HOXA11-AS promotes the proliferation HCC cells by epigenetically silencing DUSP5

**DOI:** 10.18632/oncotarget.22723

**Published:** 2017-11-27

**Authors:** Bin Liu, Jing Li, Xiaoling Liu, Min Zheng, Ye Yang, Qian Lyu, Li Jin

**Affiliations:** ^1^ Department of Medical Oncology, Sichuan Cancer Hospital & Institute, Chengdu 610041, Sichuan Province, China; ^2^ Department of General Medicine, Sichuan Cancer Hospital & Institute, Chengdu 610041, Sichuan Province, China; ^3^ State Key Laboratory of Biotherapy, West China Medical School, Sichuan University, Chengdu 610041, Sichuan Province, China; ^4^ Department of Pathology, Nanjing First Hospital, Nanjing Medical University, Nanjing 210000, Jiangsu Province, China

**Keywords:** HCC, Lnc-RNA HOXA11-AS, proliferation, EZH2, DUSP5

## Abstract

Hepatocellular carcinoma has been identified as the fifth most common cancer in men and the ninth in women worldwide. Despite many efforts have been made in recent years, the overall survival rate of patients with hepatocellular carcinoma still remain unsatisfied. Therefore, exploring the mechanisms underlying the progression of hepatocellular carcinoma is essential for developing novel treatments to improve patient prognosis. HOXA11-AS, transcribed from the opposite strand of the protein-coding gene HOXA11, has been identified to be associated with the malignant characteristics of several cancers. However, the biological role and molecular mechanism of HOXA11-AS in hepatocellular carcinoma still need to be further investigated. In the current study, the expression of HOXA11-AS in the hepatocellular carcinoma cell lines and tissues was measured by quantitative real-time PCR. Loss-of-function and gain-of-function approaches were applied to investigate the proliferative function of HOXA11-AS in hepatocellular carcinoma cells. Results from flow cytometric analysis of apoptosis and cell cycle distribution revealed that HOXA11-AS promoted hepatocellular carcinoma cells proliferation through regulating cell cycle and apoptosis. Gene chip technology and quantitative real-time PCR confirmed that DUSP5 was a downstream target of HOXA11-AS. RNA immune co-precipitation assays, RNA pull-down and Chromatin immunoprecipitation assays confirmed that HOXA11-AS could recruit EZH2 to the promoter region of DUSP5, which therefore suppressed the transcription of DUSP5. Collectively, these findings revealed that HOXA11-AS functions as an oncogene in hepatocellular carcinoma through interacting with polycomb-repressive complex2.

## INTRODUCTION

Hepatocellular carcinoma (HCC), occupying 90% of liver cancer, has been identified as the fifth most common cancer in men and the ninth in women worldwide [1]. Due to invasion, metastasis, frequent recurrence and chemo-/radio-resistance, the survival rate of HCC patients still remains unsatisfied. Therefore, it is vital to investigate the mechanism underlying HCC initiation and progression so as to find new targets for HCC.

Currently, accumulating evidences have recognized that long non-coding RNAs (lncRNAs), a class of non-coding RNA with larger than 200nt in length, have been identified as tumor suppressor or oncogene through functioning as gene regulators for regulation of multiple biological processes including proliferation, differentiation, apoptosis, tumorigenesis and metastasis or biomarkers for diagnosis of multiple neoplastic diseases, predicting survival and recurrence [2–9]. Over the past several years, the studies about lncRNAs have risen rapidly. For instance, previous studies demonstrated exosomal long noncoding RNA CRNDE-h as a novel serum-based biomarker for diagnosis and prognosis of colorectal cancer [10]. Yu et al. revealed that lnc-RNA linc00261 suppressed gastric cancer progression via promoting Slug degradation [11]. HOXA11-AS, transcribed from the opposite strand of the protein coding gene HOXA11, was first identified in a mouse embryonic cDNA library using a probe from the sense HOXA11 cDNA sequence [12, 13]. It has been shown that HOXA11-AS has the ability to repress HOXA11 mRNA expression by transcriptional interference, which is essential for embryo implantation and endometrial development [14, 15]. Abnormal expression of HOXA11-AS may be associated with the malignant characteristics of several cancers [13, 16–19]. However, the function of HOXA11-AS in HCC has not been elucidated.

The current study aimes to measure HOXA11-AS expression in HCC tissues and cells, investigate the proliferation function of HOXA11-AS levels in HCC cells, and determine whether HOXA11-AS can serve as a viable target for treating HCC.

## RESULTS

### LncRNA-HOXA11-AS is up-regulated in HCC tissues and HCC cells

**Figure 1:**
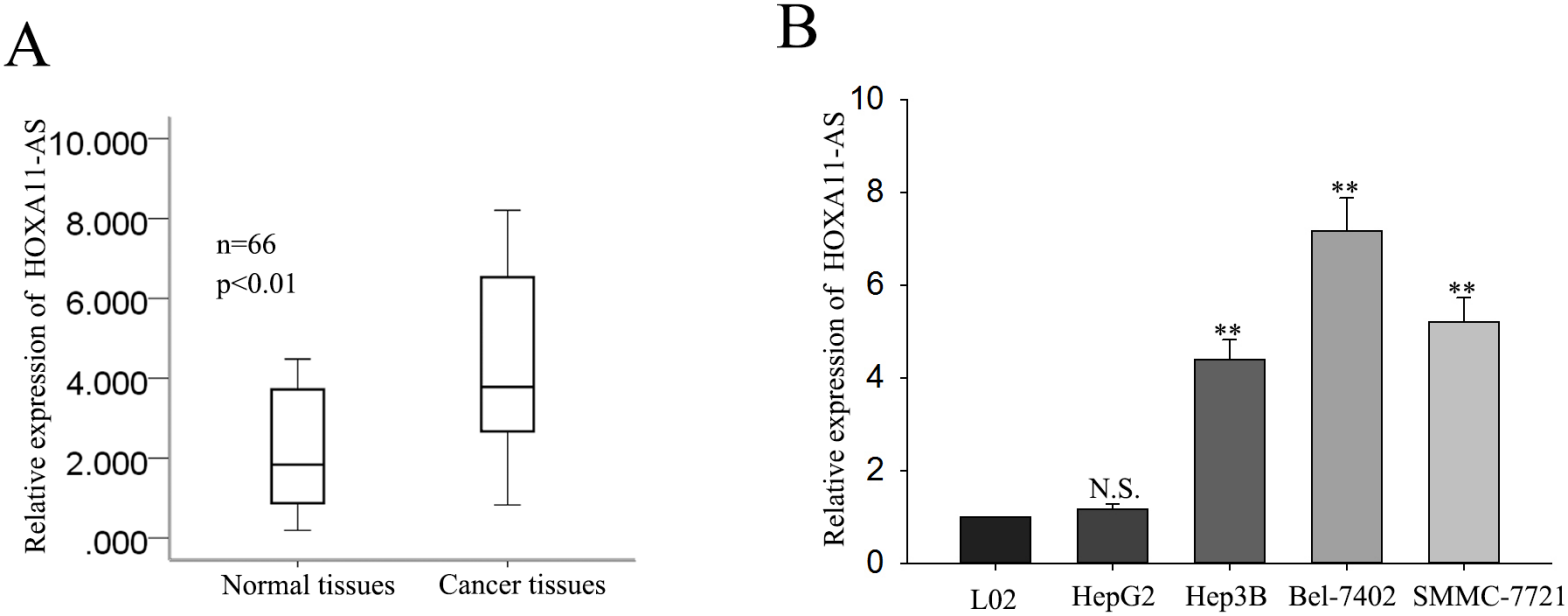
LncRNA-HOXA11-AS is up-regulated in HCC tissues and HCC cells **(A)** The level of HOXA11-AS in 66 HCC tissues and corresponding normal tissues were measured by qRT-PCR. **(B)** The level of HOXA11-AS in four HCC cell lines (HepG2, Hep3B, Bel-7402, SMMC-7721) and a normal liver epithelium cell line L02 were detected by qRT-PCR. All data were represented as the mean ± S.D. from three independent experiments. The p-value represents the comparison between groups (^*^p < 0.05, ^**^p < 0.01).

In order to explore the function of HOXA11-AS in HCC, we first employed the qRT-PCR to measure the expression level of HOXA11-AS in 66 pairs HCC tissues. As shown in Figure 1A, the level of HOXA11-AS was significantly increased in HCC tissues, compared with that in corresponding normaltissues. Next, we detected the level of HOXA11-AS in four HCC cell lines (HepG2, Hep3B, Bel-7402, SMMC-7721 HCC cell lines) and a normal liver epithelium cell line L02. In comparison to L02 cells, HCC cells exhibited significantly higher levels of HOXA11-AS expression except for HepG2 cells (Figure 1B). These results indicated that HOXA11-AS might be involved in the progression of the HCC.

### LncRNA-HOXA11-AS is correlated with the clinicopathological features and overall survival of HCC patients

**Figure 2:**
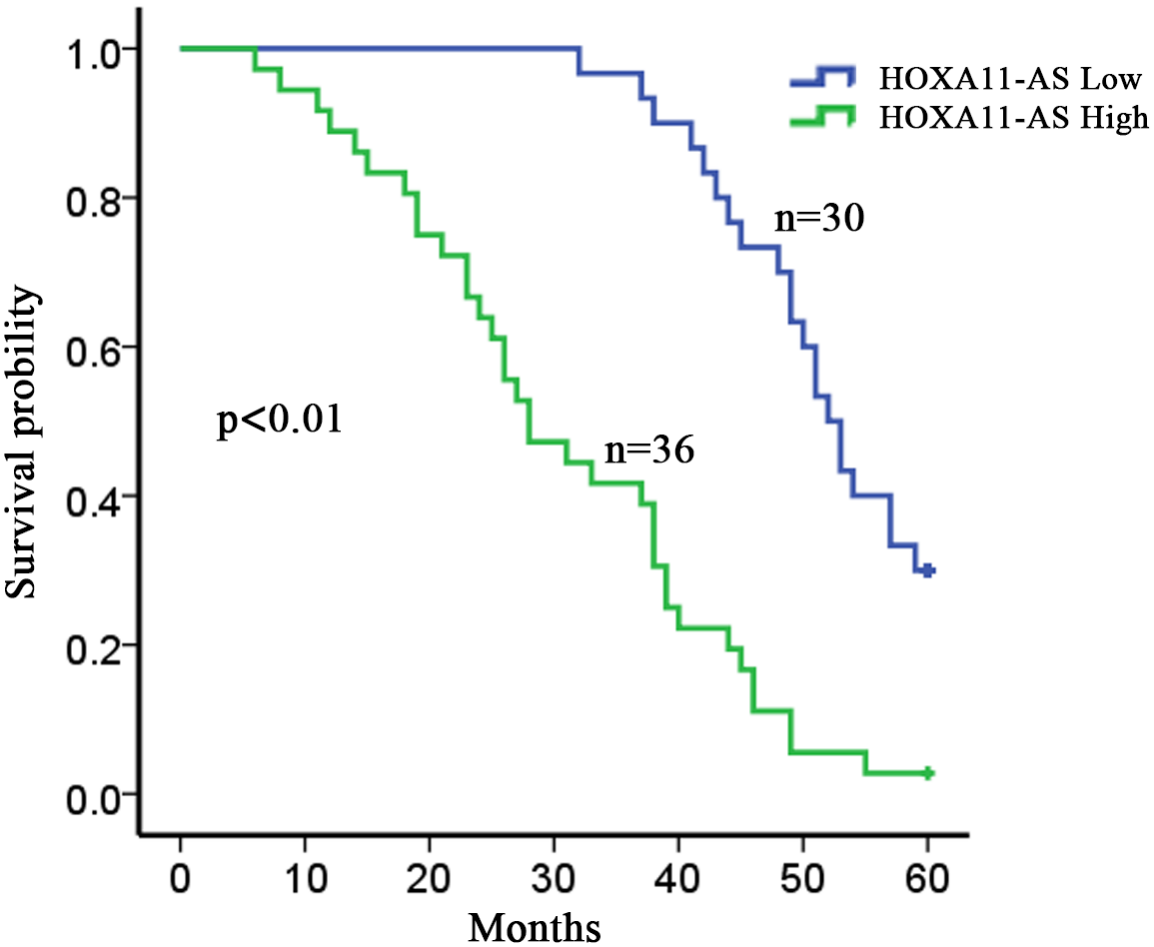
LncRNA-HOXA11-AS is correlated with the clinicopathological features and overall survival of HCC patients The overall survivals in 66 HCC patients were represented by Kaplan-Meier curves. p=0.000.

Then, we assessed the correlation of HOXA11-AS expression with the clinicopathological parameters of 66 patients diagnosed with HCC. The median value of HOXA11-AS in all HCC tissues was used as a cutoff value, and all samples were divided into two groups (high expression group n=36 vs. low expression group n=30). As presented in Table 1, high level of HOXA11-AS expression was significantly correlated with vascular invasion (p=0.000), cirrhosis (p=0.003), tumor size (p=0.002) and edmindson grade (p=0.000), but it had no significant correlation with sex, age, HbsAg, ALT and AFP (p>0.05). Furthermore, Kaplan–Meier method analysis (log-rank test) was performed to determine the association between HOXA11-AS expression and overall survival of patients. As shown in Figure 2, patients with high level of HOXA11-AS expression had a significantly shorter overall survival time than those with low level of HOXA11-AS (P < 0.05).

**Table 1:** Correlation between lnc-HOXA11-AS expression and clinical features. (n=66)

Variable	Lnc-HOXA11-AS Expression		P-value
	low	high	
**Age**			
<60	16	23	0.269
≥60	14	13	
**Gender**			
Male	28	32	0.428
Female	2	4	
**HbsAg**			
Negative	5	3	0.256
Positive	25	33	
**ALT**			
≤45	26	27	0.191
>45	4	9	
**AFP**			
≤13.6	3	1	0.241
>13.6	27	35	
**Vascular invasion**			
Absent	14	3	0.000
Present	16	33	
**Cirrhosis**			
Absent	12	3	0.003
Present	18	33	
**Tumor size**			
≤5	27	20	0.002
>5	3	16	
**Edmindson grade**			
I-II	24	5	0.000
III-IV	6	31	

Low/high by the sample median. Pearson χ^2^ test. P<0.05 was considered statistically significant.

### Dysregulated lncRNA-HOXA11-AS influences proliferation of HCC cells

**Figure 3:**
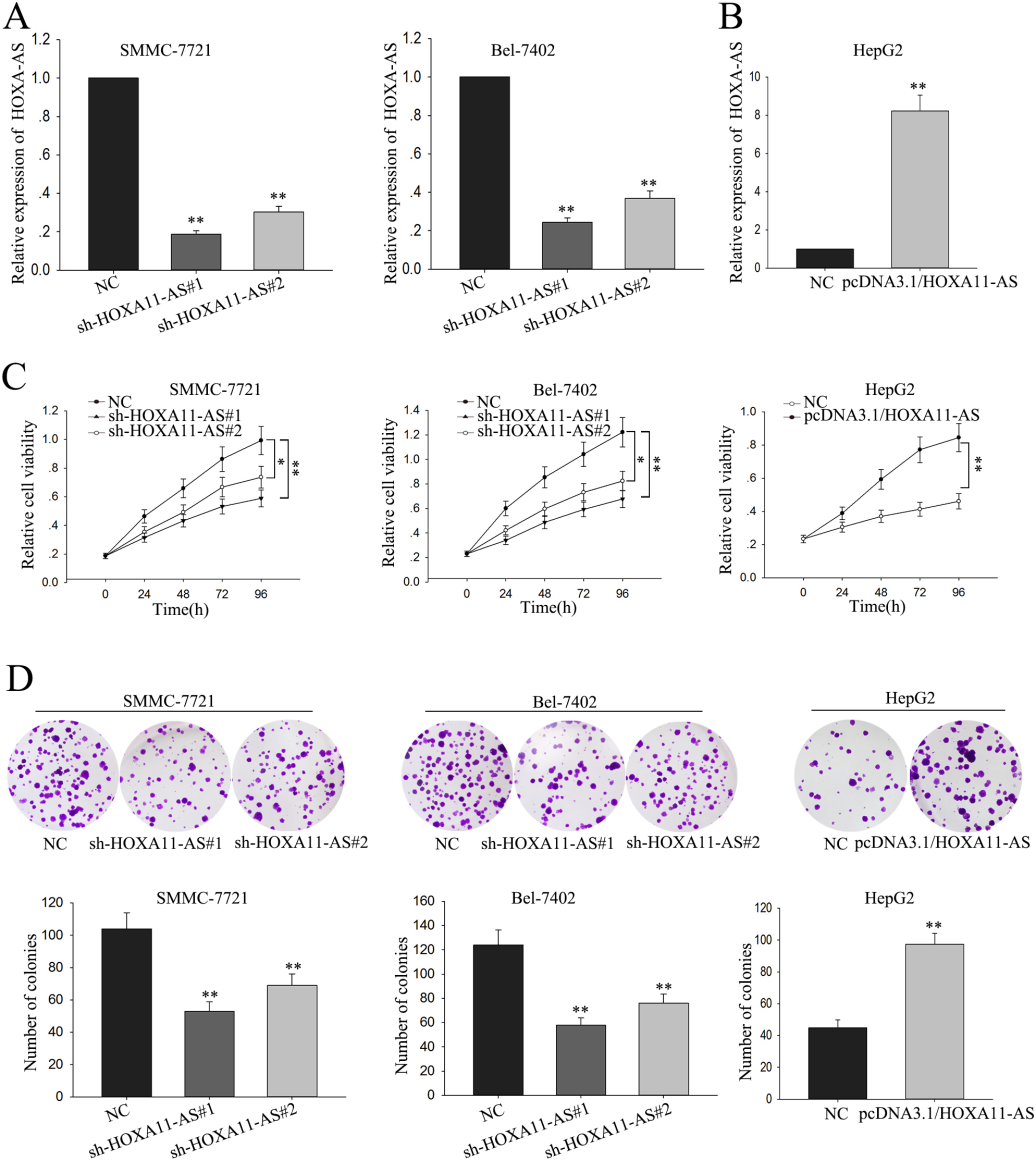
Overexpression of lncRNA-HOXA11-AS influences proliferation of HCC cells **(A-B)** SMMC-7721 cells and Bel-7402 cell were transfected with sh-HOXA11-AS using shRNA as a negative control (NC); HepG2 cells were transfected with pcDNA3.1/HOXA11-AS, using pcDNA3.1 as a negative control (NC). **(C)** MTT was performed to assess the biological function of HOXA11-AS on the cell viability of HCC cells. **(D)** Colony-formation assays were performed to measure the effect of HOXA11-AS on HCC cell proliferation. All data were represented as the mean ± S.D. from three independent experiments. The p-value represents the comparison between groups (^*^p < 0.05, ^**^p < 0.01).

To investigate the function of HOXA11-AS on the proliferation of HCC cells, SMMC-7721 cells and Bel-7402 cells were transfected with sh-HOXA11-AS (sh-HOXA11-AS#1 and sh-HOXA11-AS#2) using shRNA as a negative control (NC); HepG2 cells were transfected with pcDNA3.1/HOXA11-AS, using pcDNA3.1 as a negative control (NC). Satisfactory transfection efficiency was obtained at 48 hours post-transfection (Figure 3A and 3B, p<0.01). As shown in Figure 3C, MTT assays revealed the weakened proliferation ability of SMMC-7721cells and Bel-7402 cells transfected with sh-HOXA11-AS#1 or sh-HOXA11-AS#2, compared with NC-transfected cells; and on the contrary, an enhanced proliferation ability of pcDNA3.1/HOXA11-AS -transfected HepG2 cells in comparison to NC-transfected cells (p<0.01). In consistent with the results of MTT, colony formation assay revealed that the down-regulation of HOXA11-AS in SMMC-7721 cells and Bel-7402 cells decreased the colony formation rate, and vice versa in HOXA11-AS-transfected HepG2 cells (Figure 3D, p<0.01). The findings referred above indicated that HOXA11-AS could facilitate the proliferation of HCC cells.

### Knockdown of lncRNA-HOXA11-AS causes cell cycle arrest at G1 phase and promotes cell apoptosis

**Figure 4:**
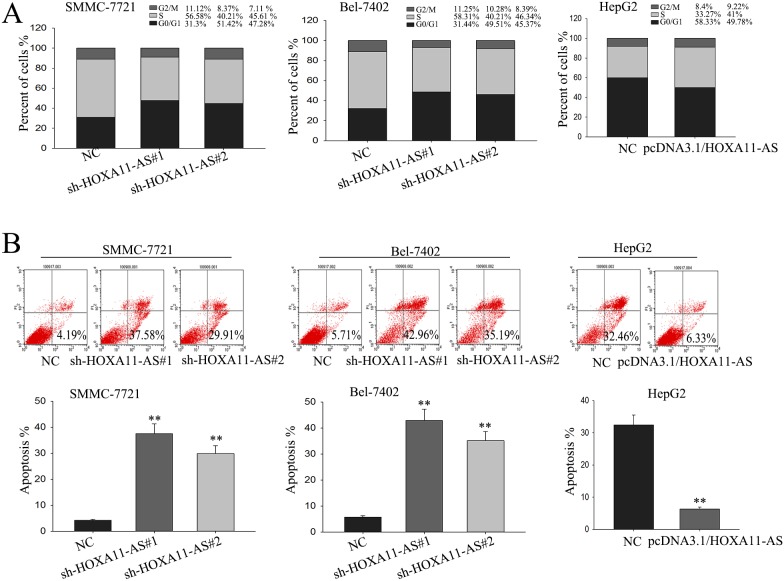
Knockdown lncRNA-HOXA11-AS causes cell cycle arrest at G1 phase and promotes cell apoptosis **(A** and **B)** Flow cytometric analysis of cell cycle distribution (A) and apoptosis (B) were utilized to determine the effect of HOXA11-AS on cell cycle and apoptosis rate. All data were represented as the mean ± S.D. from three independent experiments. The p-value represents the comparison between groups (^*^p < 0.05, ^**^p < 0.01).

To further explore the pro-proliferation mechanism of HOXA11-AS, flow cytometric analysis of apoptosis and cell cycle distribution were utilized. As shown in Figure 4A, knockdown of HOXA11-AS in SMMC-7721 cells and Bel-7402 cells significantly caused cell cycle arrest at G1, whereas forced expressed HOXA11-AS promoted cell from G1 phase to S phase. Then, flow cytometric analysis of apoptosis was performed to detect the function of HOXA11-AS on apoptosis in HCC cells. As presented in Figure 4B, knockdown of HOXA11-AS significantly increased the apoptosis rate of SMMC-7721 cells and Bel-7402 cells; on the contrary, compared with negative controls, forced expression of HOXA11-AS induced an obviously decrease in apoptosis rate in HepG2 cells. These data indicated that HOXA11-AS contributed to the proliferation ability of HCC cells, which might be attributed to its influence on cell cycle and apoptosis.

### DUSP5 was identified as a potential downstream target gene of HOXA11-AS in HCC cells

**Figure 5:**
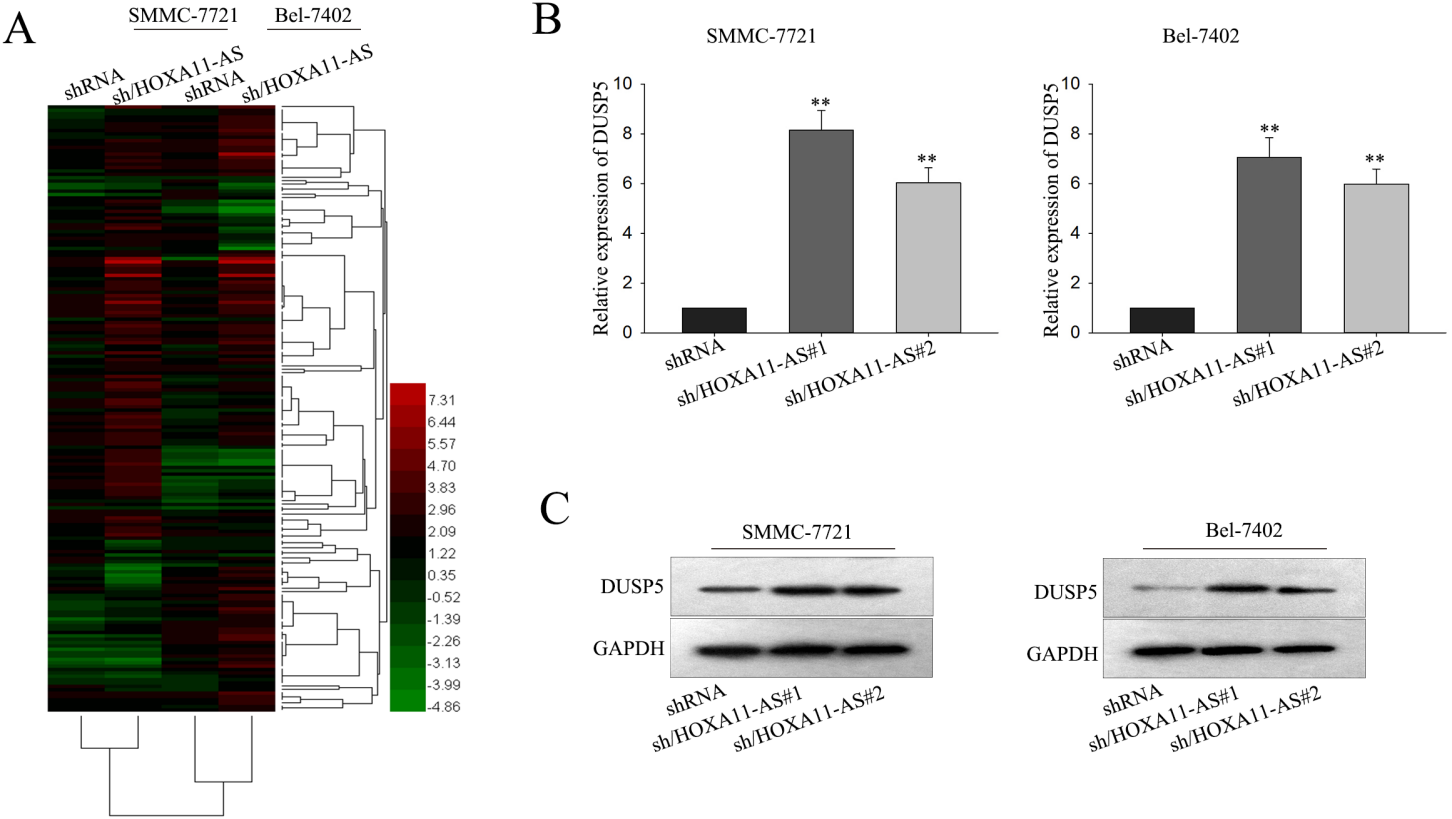
DUSP5 was identified as potential downstream target gene of HOXA11-AS in HCC cells **(A)** Gene chip technology was used to detect the differentially expressed genes after knockdown HOXA11-AS in SMMC-7721 and Bel-7402 cells. **(B** and **C)** RT-qPCR and western blot were applied to verify the level of DUSP5 in response to HOXA11-AS1 knockdown. All data were represented as the mean ± S.D. from three independent experiments. The p-value represents the comparison between groups (^*^p < 0.05, ^**^p < 0.01).

Besides characterizing the function of HOXA11-AS on HCC cells, we also sought to explore in further detail the mechanism by which HOXA11-AS exerted its functions. It had been documented that lncRNAs could exert their functions through regulating oncogenes or tumor suppressor genes. To further investigate the possible downstream targets of HOXA11-AS, we employed gene chip technology to detect the differentially expressed genes after knockdown of HOXA11-AS in SMMC-7721 and Bel-7402 cells. And the results showed that tumor suppressor genes DUSP5 was increased most significantly (Figure 5A). Then, we selected DUSP5 as the subsequent study object. RT-qPCR and western blot were applied to accurately verify the level of DUSP5 in cells and we found that DUSP5 was significantly increased in HOXA11-AS-knockdown SMMC-7721 and Bel-7402 cells (Figure 5B) and western blot assay further confirmed the result (Figure 5C). The above results suggested that DUSP5 might be the downstream gene of HOXA11-AS.

### LncRNA-HOXA11-AS recruits EZH2 and suppresses the expression of DUSP5

**Figure 6:**
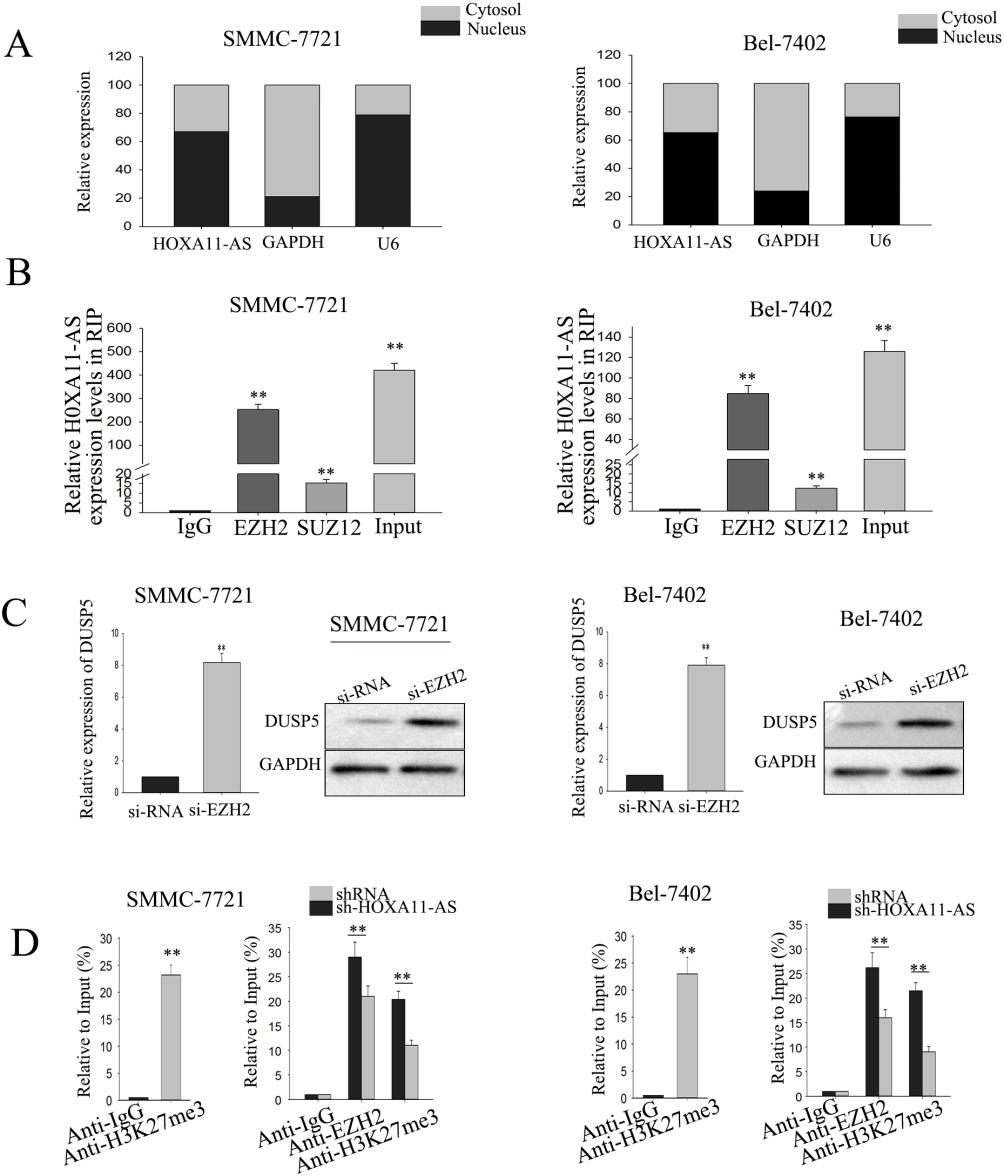
LncRNA-HOXA11-AS recruits EZH2 and suppresses the expression of DUSP5 **(A)** RT–qPCR was performed to measure the level of HOXA11-AS1 in nuclear and cytoplasmic fractions of SMMC-7721 and Bel-7402. **(B)** RIP assay were performed to confirmed the combination of HOXA11-AS1 and EZH2. **(C)** The level of DUSP5 both in mRNA and protein level were detected in response to EZH2 silenced. **(D)** ChIP assays were performed to H3K27me3 status of the promoter region of DUSP5 with or without knockdown HOXA11-AS. All data were represented as the mean ± S.D. from three independent experiments. The p-value represents the comparison between groups (^*^p < 0.05, ^**^p < 0.01).

Accumulating documents have identified that many lncRNAs function as competing endogenous (ce) RNAs by serving as sponges that bind and sequester away miRNAs [22–24]. To exam whether HOXA11-AS exerted its function in HCC cells through functioning as a ceRNA, we first measured the percentage of HOXA11-AS in the cytoplasmic and nuclear fractions of SMMC-7721 and Bel-7402 cells because its locating in cytoplasmic is a prerequisite for functioning as a ceRNA. As shown in Figure 6A, RT–qPCR of nuclear and cytoplasmic fractions of SMMC-7721 and Bel-7402 cells presented that HOXA11-AS was located in the nuclear, providing evidence that HOXA11-AS exerted its function in HCC not in a ceRNA manner. It had been described that HOXA11-AS could interact with PRC2 [18]. Therefore, we detected the relationship between HOXA11-AS and PRC2. As shown in Figure 6B and Supplementary Figure 1C, RIP and RNA pulldown assay confirmed the combination of HOXA11-AS with EZH2 (P<0.01). Then, through down-regulating EZH2 in SMMC-7721 and Bel-7402 cells (Supplementray Figure 1B), we found that the mRNA and protein levels of DUSP5 were significantly increased (Figure 6C). Additionally, as shown in Figure 6D, ChIP assay revealed that HOXA11-AS could recruit EZH2 to the promoter region of DUSP5, which therefore suppressed the transcription of DUSP5. These findings indicated that HOXA11-AS repressed its target through interacting with PRC2.

### The oncogenic function of lncRNA-HOXA11-AS in HCC cells is dependent on targeting DUSP5

**Figure 7:**
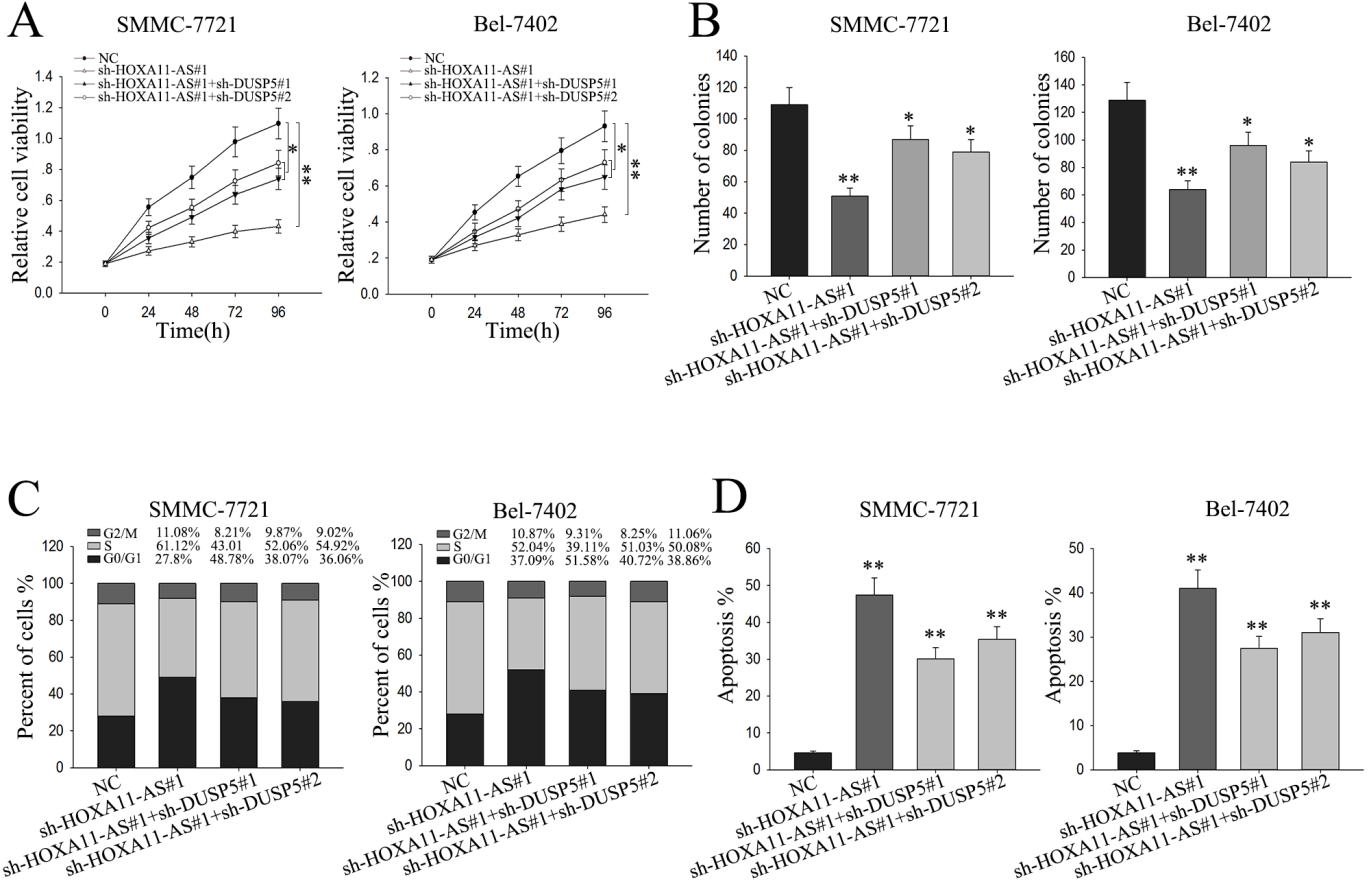
The oncogenic function of lncRNA-HOXA11-AS in HCC cells is dependent on targeting DUSP5 **(A)** MTT was performed to assess the cell viability of HCC cells co-transfected with shRNA, sh-HOXA11-AS1 or sh-DUSP5. **(B)** Colony-formation assays were performed to measure the proliferation ability of HCC cells co-transfected with shRNA, sh-HOXA11-AS1 or sh-DUSP5. **(C** and **D)** Flow cytometry analysis were performed to assess the cell cycle percent and apoptosis rate of HCC cells co-transfected with shRNA, sh-HOXA11-AS1 or sh-DUSP5. All data were represented as the mean ± S.D. from three independent experiments. The p-value represents the comparison between groups (^*^p < 0.05, ^**^p < 0.01).

To further confirm that the function of HOXA11-AS exerted in HCC cells was mediated by DUSP5, rescue assays were applied. SMMC-7721 or Bel-7402 cells were co-transfected with shRNA, sh-HOXA11-AS and sh-DUSP5 (Supplementray Figure 1A), and MTT assay and colony formation assay were utilized to detect the cells viability and proliferation ability. As shown in Figure 7A-7B, weakened viability and proliferation ability induced by sh-HOXA11-AS was partially abolished when cells were co-transfected with sh-DUSP5. Moreover, flow cytometric analysis indicated that G0/G1-phase arrest and apoptosis increased caused by the silenced HOXA11could be abolished by silencing DUSP5 (Figure 7C and 7D). These results indicated that the effect of HOXA11-AS on progression of HCC cells was at least partially through targeting DUSP5.

### The level of DUSP5 is down-expressed in HCC tissues and is negatively correlated with lncRNA-HOXA11-AS expression level

**Figure 8:**
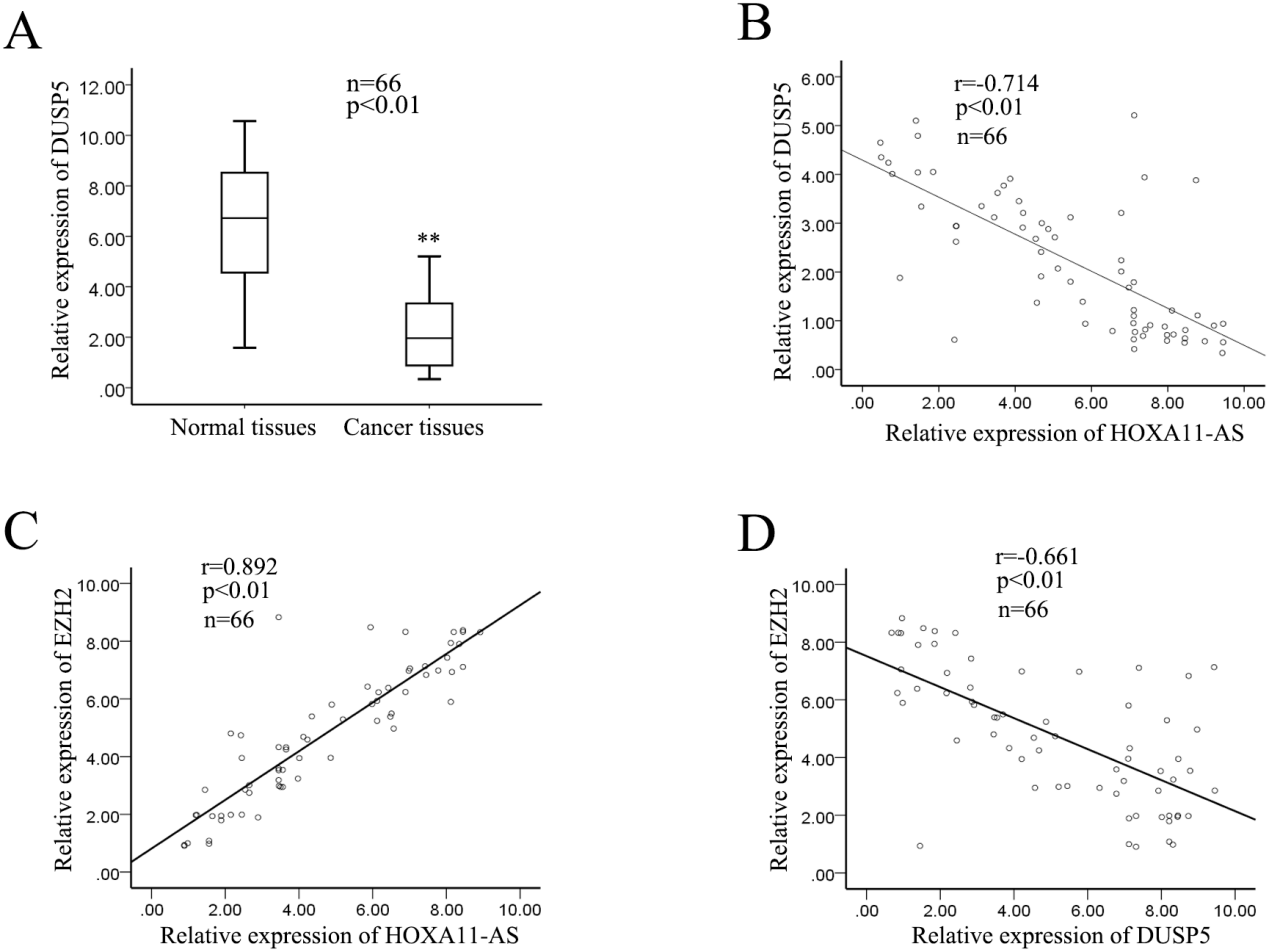
The level of DUSP5 was down-expressed in HCC tissues and is negatively correlated with lncRNA-HOXA11-AS expression level **(A)** The level of DUSP5 in 66 HCC tumor tissue samples by RT-qPCR. **(B)** The level of DUSP5 was negative correlation with the level of HOXA11-AS expression level (2-tailed Spearman’s correlation, r=-0.714, p=0.000). **(C-D)** The correlation between EZH2 and DUSP5, HOXA11-AS and EZH2 were analyzed (2-tailed Spearman’s correlation, r=-0.661, p<0.01; r=0.892, p<0.01). The p-value represents the comparison between groups (^*^p < 0.05, ^**^p < 0.01).

To further elucidate the correlations between HOXA11-AS and DUSP5 in HCC, we measured the level of DUSP5 in 66 HCC tumor tissue samples. As shown in Figure 8A, the relative level of DUSP5 expression in the tumor tissue samples was significantly lower than that in corresponding histologically normal tissues (p<0.01). What’s more, the level of DUSP5 was negatively correlated with the level of HOXA11-AS expression level (2-tailed Spearman’s correlation, r=-0.714, p=0.000; Figure 8B). Additionally, we also analyzed the expression relationship between EZH2 and DUSP5, HOXA11-AS and EZH2, and the results revealed that the level of EZH2 was negatively correlated with the level of DUSP5 (Figure 8C, 2-tailed Spearman’s correlation, r=-0.661, p<0.01), but was positively correlated with HOXA11-AS1 (Figure 8D, 2-tailed Spearman’s correlation, r=0.892, p<0.01). Collectively, these finding indicateed that HOXA11-AS facilitated the progression of HCC by targeting DUSP5.

## DISCUSSION

In the current study, we revealed that the lncRNA-HOXA11-AS was significantly increased in HCC tissues and critical for the proliferation of HCC cells. The function of lncRNA-HOXA11-AS exerted in HCC was through recruiting EZH2 and targeting DUSP5 expression.

Hepatocellular carcinoma (HCC) is the third leading cause of cancer-associated mortalities. Despite plenty basic biological researches and clinical treatment have been made, the overall survival of HCC patients is still unsatisfied. Currently, the abnormal expression of lncRNAs has been shown to involve in the pathogenesis of many cancers, providing novel therapeutic opportunities to treat cancer [25–27]. As dozens of lncRNAs have been identified as critical players in the growth and metastasis of cancer cells, attention toward lncRNAs in cancer is continuing to increase. Although accumulating researches about lncRNAs have been made, the roles of lncRNAs in HCC carcinogenesis are not well understood. Making lucubration on the molecular mechanism underlying the development and progression of HCC is favor for the exploitation of novel therapeutic targets. In the present study, we provided evidences that lncRNA-HOXA11-AS acted as an oncogene and promoted the proliferation of HCC cells by recruiting EZH2 and targeting DUSP5 expression in HCC cells.

HOXA11-AS, transcribed from the opposite strand of the protein-coding gene HOXA11, was first identified in a mouse embryonic cDNA library using a probe from the sense HOXA11 cDNA sequence [12, 13]. Abnormal expression of HOXA11-AS has been reported to be associated with the malignant characteristics of several cancers [13, 16–19]. However, the mechanism by which HOXA11-AS exerts its oncogenic functions in the tumorigenesis of HCC remains unclear. In this study, we first measured the level of HOXA11-AS in HCC tissues and HCC cells. And we found that the level of HOXA11-AS was significantly higher than that in corresponding normal-tissues. Then, we comprehensively investigated the functions of HOXA11-AS in HCC cells by employing gain-of-function and loss-of-function approaches. Forced expression of HOXA11-AS facilitated the proliferation of HCC cells; while knockdown of HOXA11-AS inhibited the proliferation of HCC cells.

LncRNAs can guide and recruit DNA, histone protein modification enzymes, or transcription factors to specific genomic loci, leading to inactivation of tumor suppressors or activation of oncogenes [28]. It has been reported that HOXA11-AS interacts with PRC2 [18]. In present study, we found that HOXA11-AS could bind to EZH2. EZH2 is a critical component of the polycomb-repressive complex2 (PRC2) contributing to many essential biological processes. DUSP5 is a tumor suppressor and has been reported to be down-regulated in certain cancers [29–31]. In our study, we identified that DUSP5 was a target of HOXA11-AS in HCC cells. We found that HOXA11-AS could recruit EZH2 to the promoter of DUSP5 and mediated the transcription suppression of DUSP5. And gain-of-function and loss-of-function revealed that DUSP5 functionedas a tumor suppressor in HCC cells. What’s more, rescue assays further confirmed that DUDP5 mediated the pro-proliferation and anti-apoptosis function of HOXA11-AS. Last, we examined the level of DUSP5 in HCC tissues and found that the level of DUSP5 was negatively correlated with the level of HOXA11-AS.

In general, our data suggested that lncRNA-HOXA11-AS could promote the proliferation of HCC cells by recruiting EZH2 and suppressing the expression of the tumor suppressor DUSP5. Our study provided new insight into the mechanisms underlying the proliferation mechanism of HCC cells, which might be targeted for therapeutic benefits.

## MATERIALS AND METHODS

### Patients

All informed consent was obtained from patients. HCC tissue samples were obtained from 66 patients (60 males and 6 females) diagnosed with HCC who had undergone a routine hepatic resection in the Sichuan Cancer Hospital & Institute between 2006 and 2012. Adjacent normal tissues, which were defined as normal in the results, were obtained 2 cm distal from the HCC tissue. These tissues were divided into two groups according to the Edmindson grade: I–II HCC group (n=29) and grades III–IV HCC group (n=37). None of the patients had received preoperative radiotherapy or chemotherapy prior to surgical resection. Histological diagnosis and differentiation were evaluated independently by three pathologists according to the WHO classification system. The project protocol was approved by the Institutional Ethics Committee of Sichuan Cancer Hospital & Institute prior to the initiation of the study.

### Cell lines

For routine culture, HepG2, Hep3B, Bel-7402, SMMC-7721 HCC cell lines and a normal liver epithelium cell line L02 were purchased from the Shanghai Cell Collection (Shanghai, China) and were maintained in high-glucose DMEM (Gibco, 8113035) medium with 10% fetal bovine serum (10% FBS), 100 U/mL penicillin, and 100 mg/mL streptomycin in humidified air at 37°C with 5% CO2.

### Quantitative real–time PCR

Total RNA was extracted from tissues and cancer cells using RNAiso Plus (TaKaRa, 09108B). cDNA synthesis was performed according to the manufacturer’s instructions (RR047A, TaKaRa, Da Lian, China), and qRT-PCR was performed with SYBR Premix Ex Taq II (TaKaRa, DRR081A) using a LightCycler system (Roche, Basel, Switzerland). The PCR reaction conditions for all of the assays were 94 °C for 30 seconds, followed by 40 cycles of amplification (94 °C for 5 seconds, 60 °C for 30 seconds and 72 °C for 30 seconds). The HOXA11-AS, EZH2 and DUSP5 were calculated with the 2-ΔΔCt method, which was normalized to GAPDH. All assays were performed in triplicate. The primer were provided as following: HOXA11-AS forward: 5’-TGCCAAGTTGTACTTACTACGTC-3’, and reverse:5’-GTTGGAGGAGTAGGAGTATGTA-3’; EZH2 forward:5-GGACTAGTGGAGAAGGTGCG-3’ reverse:5’-GGGCGCTGCCCATCATCATG-3’ ; DUSP5 forward:5’-TCCCTGACTTCTAGCCCTGT-3’ reverse:5’TTTAGCAGGATGTGGCCGTT-3’; GAPDH forward:5’-GGGAGCCAAAAGGGTCAT-3’ reverse:5’-GAGTCCFTTCCACGATACCAA-3’. The expression levels were relative to the fold change of the corresponding controls, which were defined as 1.0.

### Microarray analysis

The platform was the Agilent Human mRNA Microarray, Release 21.0 (Design ID: BC13137). The total RNA was quantified by the NanoDrop ND-2000 (Thermo Scientific, Waltham, USA) and the RNA integrity was assessed using Agilent Bioanalyzer 2100 (Agilent Technologies). The sample labeling, microarray hybridization and washing were performed according to the manufacturer’s standard protocols. Briefly, total RNAs were dephosphorylated, desaturated and labeled with Cyanine-3-CTP. After purification, the labeled RNAs were hybridized onto the microarray. After being washed, the arrays were scanned with the Agilent Scanner G2505C (Agilent Technologies). Feature Extraction software (version10.7.1.1; Agilent Technologies) was used to analyze array images to get the raw data. Genespring software (version 13.1; Agilent Technologies) was used to carry out the basic analysis with the raw data. The raw data were normalized with the quantile algorithm. Differentially expressed mRNAs were then identified through fold change as well as P-value calculated by using a two-sided t-test on a single mRNA basis. The ratios of the P-value < 0.05 were generally accepted as true.

### Cell viability

Cell viability was assessed via 3-(4,5-dimethylthiazol-2-yl)-2, 5-diphenyl-trtrazolium bromide (MTT) assay. 5 × 103 cells/well were seeded in a 96-well flat-bottomed plate for 24 h, then transfected with indicated vectors and cultured in normal medium. At 0, 24, 48, 72 h and 96h after transfection, the MTT solution (5 mg/ml, 20 μl) was added to each well. Following incubation for 4 h, the media was removed and 100 μl DMSO was added to each well. The relative number of surviving cells was assessed by measuring the optical density (O.D.) of cell lysates at 560 nm. All assays were performed in triplicate.

### Colony formation assay

Cells (500 cells/well) were plated in 6-well plates and incubated in normal medium at 37°C. After two weeks, the cells were fixed and stained with 0.1% crystal violet in methanol. The number of visible colonies was counted manually.

### Flow cytometric analysis of apoptosis

Apoptosis was performed using flow cytometric analyses with Annexin V: FITC Apoptosis Detection Kits (BD Biosciences, USA), according to the manufacturer’s instructions. All samples were assayed in triplicate.

### Flow cytometric analysis of cell cycle distribution

Cells were collected directly or 48 hours after transfection and washed with ice-cold phosphate-buffered saline (PBS), and fixed with 70% ethanol overnight at -20°C. Fixed cells were rehydrated in PBS for 10 minutes and incubated in RNase A (1mg/ml) for 30min at 37°C, then the cells were subjected to PI/RNase staining followed by flow cytometric analysis using a FACScan instrument (Becton Dickinson, Mountain View, CA) and Cell Quest software (Becton Dickinson, San Jose, CA) as described previously [20].

### RNA immune co-precipitation (RIP) assays

RNA immunoprecipitation was performed using thermo fisher RIP kit (Thermo, USA) based on the manufacturer’s protocol. Cells at 80–90% confluency were scraped off and lysed in complete RIP lysis buffer. Then, 100 μl of whole cell extract was incubated with RIP buffer containing magnetic beads conjugated with human anti-EZH2 antibody (Cell Signaling, USA), anti-SUZ12 antibody (Cell Signaling, USA), anti-EED antibody (Cell Signaling, USA) negative control normal mouse IgG (Millipore).

### ChIP assay

Chromatin immunoprecipitation (ChIP) assays were performed using the Millipore ChIP Assay Kit (catalogno.: 17-295) according to the introductions. And antibodies for H3 (tri methylK27; ab6002) and EZH2 were purchased from Abcam (USA).

### Subcellular fractionation location

The separation of nuclear and cytosolic fractions was performed using the PARIS Kit (Life Technologies, Carlsbad, CA, USA) according to the manufacturer’s instructions.

### RNA pulldown assays

HOXA11-AS RNAs were *in vitro* transcribed using T7 RNA polymerase (Ambio Life, Cheyenne, WY, USA), which were then purified using the RNeasy Plus Mini Kit (Qiagen) and treated with RNase-free DNase I (Qiagen). Transcribed RNAs were biotin-labeled with the Biotin RNA Labeling Mix (Ambio Life). Positive, negative and Biotinylated RNAs were mixed and incubated with HCC cell lysates. Magnetic beads were added to each binding reaction, followed by incubation at room temperature. Then, the beads were washed with washing buffer. The eluted proteins were detected by western blot analysis.

### Western blotting

The procedure was described by Tang et al.[21]. All antibodies (EZH2 (1: 2000 dilution), DUSP5 (1: 2000 dilution), GAPDH (1:3000)) were purchased from Abcam (USA). Membranes were blocked with 5% (v/v) milk and incubated with the primary antibodies in 5% (w/v) bovine serum albumin (BSA) at 4°C overnight, followed by incubation with the corresponding horseradish peroxidase-linked secondary antibodies. Blots were washed for 15 min three times and the signals were detected using an enhanced chemiluminescence-detecting kit (Thermo Fisher, MA, USA) followed by exposure with Tanon 5200 Biotanon, China).

### Statistical analysis

All experiments were performed at least three times, and presented as mean ± SD. The SPSS 17.0 software (SPSS Inc., Chicago, IL, USA) was used for statistical analysis. Two group comparisons were performed with a Student t test. Multiple group comparisons were analyzed with one-way ANOVA. Long-rank test was for KaplanMeier survival analysis. All tests performed were two-sided. Statistically significant positive correlation between HOXA11-AS and DUSP5 expression levels in 66 cases of HCC tissues was analyzed by Spearman’s correlation analysis P< 0.05 or less was considered significant.

## SUPPLEMENTARY MATERIALS FIGURE


