# Supplementary material for: Long non-coding RNA HOXA11-AS promotes the proliferation HCC cells by epigenetically silencing DUSP5
Source: Oncotarget. 2017 Nov 27;8(65):109509–21. doi: 10.18632/oncotarget.22723 (PMC5752538; doi:10.18632/oncotarget.22723)
Supplement: Supplementary file 1 [file oncotarget-08-109509-s001.pdf]

Long non-coding RNA HOXA11-AS promotes the proliferation HCC cells by epigenetically silencing DUSP5

SUPPLEMENTARY MATERIALS

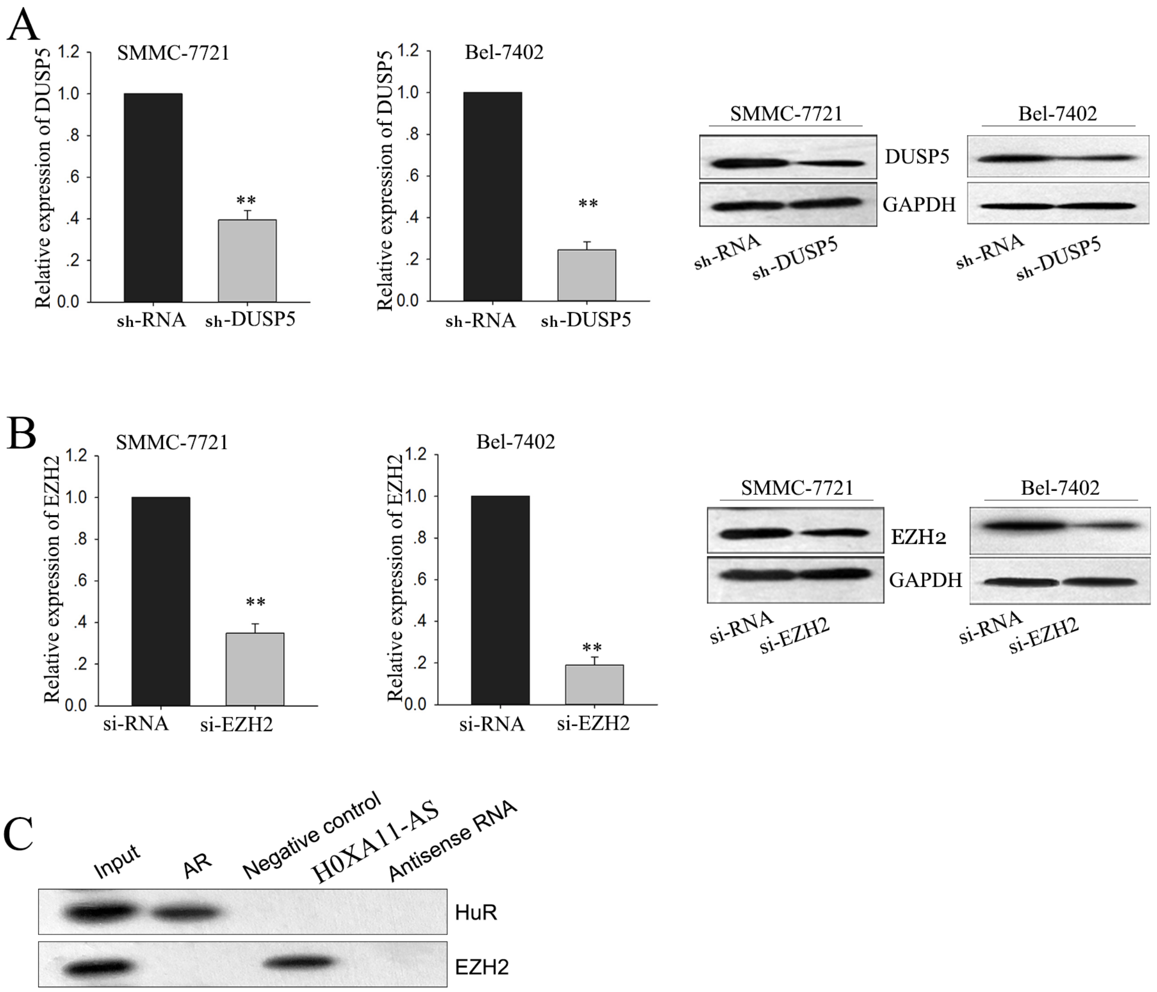

Supplementary Figure 1: (A) qRT-PCR and western blot assay were performed to detect the transfection efficiency of sh-RNA and sh-DUSP5. **(B)** qRT-PCR and western blot assay were performed to detect the the transfection efficiency of si-EZH2. **(C)** RNA pulldown was performed to determine the combination between HOXA11-AS and EZH2. All data were represented as the mean ± S.D. from three independent experiments. The p-value represents the comparison between groups ($^{*}p < 0.05$, $^{**}p < 0.01$).
